# Hypoxia Triggers BNIP3/BNIP3L-Associated Mitophagy in HT22 Immortalized Hippocampal Neurons: A Temporal Descriptive Study

**DOI:** 10.3390/biology15181659

**Published:** 2026-09-19

**Authors:** Mingze Zheng, Xinqin Liu, Yang Zhou

**Affiliations:** 1 School of Basic Medicine, Fourth Military Medical University, Xi’an 710032, China; zhengmingze2024@163.com; 2 Department of Occupational and Environmental Health, The Ministry of Education Key Laboratory of Hazard Assessment and Control in Special Operational Environment, School of Public Health, Fourth Military Medical University, Xi’an 710032, China; 3 Shanxi Key Laboratory of Environment Health Hazard Assessment and Protection, School of Public Health, Fourth Military Medical University, Xi’an 710032, China

**Keywords:** hypoxia, mitophagy, BNIP3/BNIP3L, hippocampal neurons, temporal dynamics, mitochondrial fragmentation

## Abstract

Hypoxia poses a significant threat to hippocampal neurons, but the temporal dynamics of mitophagy pathways in neuronal models remain incompletely characterized. This study aims to delineate time-dependent mitochondrial structural damage, fragmentation, and mitophagic responses in hippocampal neuronal cells under sustained hypoxia and to explore the key mitophagy receptors involved. Murine HT22 immortalized hippocampal neuronal cells were exposed to 1% O_2_ for 6–72 h. Mitochondrial integrity was assessed using Western blot and transmission electron microscopy. Mitochondrial fragmentation was quantified via HSP60 intensity, mitochondrial length, and number of fragments per cell using immunofluorescence and MitoTracker live-cell labeling. Mitophagic activity was evaluated using LC3B puncta quantification, LC3B–Tom20 colocalization, and mitochondria–lysosome proximity via LysoTracker co-labeling. The transcriptional responses of five mitophagy-related genes (*Bnip3*, *Bnip3l*, *Pink1*, *Fundc1*, and *Atg5*) were screened using RT-qPCR, with protein validation by Western blot and immunofluorescence. Prolonged hypoxia progressively reduced Tim23 and Tom20, with ultrastructural damage and mitochondrial fragmentation seen. Mitophagy-associated phenotypes were evident, as shown by increased LC3B puncta, LC3B–Tom20 colocalization, and mitochondria–lysosome proximity. *Bnip3* and *Bnip3l* showed the earliest and most pronounced transcriptional upregulation, with BNIP3 and BNIP3L protein levels elevated from 12 to 48 h. BNIP3 and BNIP3L intensities increased after 48 h, exhibiting granular and filamentous morphologies, respectively. This study provides a temporal framework for hypoxia-induced mitophagy in hippocampal neurons, identifying BNIP3/BNIP3L as early and prominent responders, which may inform therapeutic targeting in hypoxic brain injury.

## Simple Summary

This study characterizes the temporal progression of hypoxia-induced mitophagy in HT22 immortalized murine hippocampal neuronal cells exposed to 1% O_2_ for 6–72 h. We observed a progressive degradation of mitochondrial proteins Tim23 and Tom20, mitochondrial fragmentation, increased LC3B puncta, and increased spatial proximity between the mitochondria and lysosomes. RT-qPCR screening of five mitophagy-related genes revealed that *Bnip3* and *Bnip3l* exhibited the earliest (6 h) and most pronounced transcriptional upregulation relative to *Pink1*, *Fundc1*, and *Atg5*, which was paralleled by BNIP3 and BNIP3L protein elevation from 12 to 48 h. Immunofluorescence further revealed increased mean fluorescence intensities of BNIP3 and BNIP3L at 48 h, which exhibited distinct granular (BNIP3) and filamentous (BNIP3L) morphologies. These descriptive findings provide a temporal framework for understanding hypoxia-associated mitochondrial clearance in a neuronal cell model.

## 1. Introduction

Hypoxia is a pervasive pathological stressor that profoundly impairs central nervous system function, with the hippocampus exhibiting particular vulnerability to oxygen deprivation due to its high metabolic demand and unique neurovascular coupling characteristics [1,2]. The hippocampus is essential for spatial and episodic memory, and its sensitivity to hypoxia is implicated in the cognitive deficits observed in conditions ranging from high-altitude exposure to cerebral ischemia and neurodegenerative diseases [1,3,4]. At the cellular level, previous research has demonstrated that hippocampal neurons are susceptible to variations in oxygen tension, manifesting as impaired synaptic plasticity, excitotoxicity, mitochondrial dysfunction, and ultimately cell death [5,6,7,8]. However, the precise mechanisms underlying hypoxia-induced hippocampal neuron dysfunction remain incompletely understood and warrant further investigation.

As the primary consumers of cellular oxygen and the main site for ATP production, mitochondria have their homeostasis disrupted by hypoxia, thereby potentially serving as key mediators of hypoxia-induced hippocampal neuron dysfunction [9,10]. Hypoxic stress triggers a cascade of mitochondrial dysfunction, including respiratory chain impairment and calcium dysregulation, as well as profound morphological alterations such as swelling, cristae disruption, and fragmentation [7,11]. These structural and functional deficits compromise cellular energy supply and can initiate cell death pathways, underscoring the critical need for quality control mechanisms that selectively remove damaged mitochondria to preserve neuronal viability [12,13]. However, the precise sequence of subcellular events linking hypoxic stress to mitochondrial damage and the cellular mechanisms engaged to cope with this damage remain incompletely characterized in hippocampal neurons.

The selective autophagic degradation of mitochondria, termed mitophagy, serves as a primary intracellular quality control system for eliminating dysfunctional mitochondria to maintain a healthy mitochondrial network [14,15]. Hippocampal neuronal injury induced by ischemic hypoxia can be attenuated by the enhancement of mitophagy [16]. PINK1/Parkin pathway-mediated mitophagy plays a crucial role in maintaining mitochondrial homeostasis following cerebral ischemia injury [17,18]. Additionally, hypoxia-associated mitophagy receptors such as BNIP3, BNIP3L, and FUNDC1 are activated in the brain following intermittent hypoxia exposure, and their upregulation has been linked to hippocampal damage and cognitive dysfunction [19]. Among these, BNIP3L/NIX, a mitophagy receptor, is degraded by proteasomes in cerebral ischemia, resulting in transient mitophagy deficiency, whereas inhibition of its degradation alleviates ischemic brain injury [20]. However, although multiple mitophagy pathways have been identified, most previous studies have used short-term or intermittent hypoxia models and focused largely on individual pathways in isolation [19,20]. Whether the PINK1/Parkin pathway and receptor-mediated pathways (e.g., BNIP3L) exhibit temporal synergy or compensation in hippocampal neurons under sustained hypoxia and whether the critical pathway changes with hypoxia duration remain unknown. Thus, the endogenous operation, temporal dynamics, and relative contributions of these pathways in hippocampal neurons under low oxygen tension merit further exploration.

To advance our understanding of these issues, we exposed HT22 immortalized murine hippocampal neuronal cells to sustained hypoxia (1% O_2_) for 6 to 72 h and performed a descriptive, multi-level temporal characterization integrating Western blot, confocal immunofluorescence, live-cell imaging, transmission electron microscopy, and RT-qPCR screening of five key genes (*Bnip3*, *Bnip3l*, *Pink1*, *Fundc1*, and *Atg5*). This study aims to construct a temporal profile of hypoxia-induced mitochondrial quality control responses in a neuronal cell model, with particular focus on the association between BNIP3/BNIP3L expression dynamics and mitophagy-related phenotypes. We emphasize that this is an observational study designed to generate hypotheses for subsequent functional investigation, rather than to establish causal mechanisms or functional outcomes.

## 2. Materials and Methods

### 2.1. Cell Culture and Hypoxia Treatment

HT22 immortalized murine hippocampal neurons (Catalog No. iCell-m020) were obtained from iCell Bioscience Inc. (Shanghai, China). HT22 cells were propagated in DMEM (Gibco, Grand Island, NY, USA) fortified with 10% fetal bovine serum (Beyotime Biotechnology, Shanghai, China) alongside 1% penicillin–streptomycin (Gibco, Grand Island, NY, USA), and incubated at 37 °C within a humidified 5% CO_2_ atmosphere. For immunofluorescence staining and confocal microscopy observation, cells were seeded in six-well plates with glass coverslips and 35 mm glass-bottom culture dishes, respectively. Cells for Western blotting and RT-qPCR analysis were cultured in 10 cm cell culture dishes. After seeding, the cells were incubated for 24 h to achieve full attachment, at which point the cell confluence reached 70–80%, and the cells were then divided into groups for subsequent treatments. For hypoxic treatment, the culture medium was pre-equilibrated inside the DWS-H85 anaerobic workstation (Don Whitley Scientific, Bingley, UK) for a minimum of 9 h prior to use; cells were then refreshed with this pre-conditioned medium and cultured within the workstation under a gas mixture of 1% O_2_, 5% CO_2_, and 94% N_2_. Cells in the normoxic control group were re-fed with fresh standard complete medium and cultured under normoxic conditions (21% O_2_, 5% CO_2_). Cells from both groups were harvested at 6, 12, 24, 48, and 72 h post-hypoxic initiation for downstream assays.

### 2.2. Western Blot Analysis

Cells were lysed in RIPA buffer containing 1 mM PMSF and a protease inhibitor cocktail (Beyotime Biotechnology, Shanghai, China). Protein concentrations were determined by BCA assay (Thermo Fisher Scientific, Waltham, MA, USA). Equal amounts of protein were resolved on 10% SDS-PAGE gels and transferred to 0.22 µm PVDF membranes (Millipore, Billerica, MA, USA). Membranes were blocked with 5% non-fat milk in TBS-T for 1 h at room temperature, then incubated overnight at 4 °C with primary antibodies against Tim23 (1:1000; BD Biosciences, San Jose, CA, USA), Tom20 (1:2000; BD Biosciences, San Jose, CA, USA), BNIP3 (1:1000; Abcam, Cambridge, UK), BNIP3L (1:500; Abcam, Cambridge, UK), and β-actin (1:2000; Abcam, Cambridge, UK). After washing, blots were incubated with HRP-conjugated secondary antibodies (1:2000; Abcam, Cambridge, UK) for 1 h at room temperature. Bands were visualized using ECL substrate (Mishu Biotech, Xi’an, Shaanxi, China). Data were collected from at least three independent experiments.

### 2.3. Immunofluorescence Staining

Cells were seeded onto glass coverslips pre-coated with 0.1 mg/mL poly-L-lysine (Sigma-Aldrich, St. Louis, MO, USA) placed in six-well plates. After cell attachment and hypoxic treatment, cells were fixed with 4% paraformaldehyde for 15 min, permeabilized with 0.3% Triton X-100 (Sigma-Aldrich, St. Louis, MO, USA) for 10 min, and blocked with 5% bovine serum albumin at room temperature for 1 h. Primary antibodies were applied overnight at 4 °C: LC3B (1:200; Cell Signaling Technology, Danvers, MA, USA), HSP60 (1:500; Cell Signaling Technology, Danvers, MA, USA), BNIP3 (1:200; Abcam, Cambridge, UK), BNIP3L (1:100; Abcam, Cambridge, UK), and Tom20 (1:100; Abcam, Cambridge, UK). Alexa Fluor 488- or 594-conjugated secondary antibodies (1:500; Abcam, Cambridge, UK) were applied for 1 h at room temperature. Antifade Mounting Medium with DAPI (Beyotime, Shanghai, China) was employed to seal slides, which were imaged via immunofluorescence microscopy (Olympus, Tokyo, Japan). Immunofluorescence images were quantitatively analyzed using ImageJ software (version 1.54p). Morphologically intact single cells were randomly selected, and the effective cellular regions were manually delineated after background fluorescence subtraction. The mean fluorescence intensity and fluorescent puncta number of target proteins in each group were quantified.

### 2.4. Mitochondrial and Lysosomal Localization Analysis

For live-cell imaging, cells cultured in glass-bottom dishes were incubated with MitoTracker Green FM (100 nM; Thermo Fisher Scientific, Waltham, MA, USA) and LysoTracker Red DND-99 (75 nM; Thermo Fisher Scientific, Waltham, MA, USA) in pre-warmed serum-free medium at 37 °C for 30 min in the dark. Discard the staining working solution, gently rinse the cells once with a small volume of prewarmed phenol red-free and serum-free DMEM, replenish sufficient fresh medium, and immediately observe under a laser scanning confocal microscope (Nikon, Tokyo, Japan). Fluorescence images were acquired with a Nikon A1plus laser scanning confocal microscope (Nikon, Tokyo, Japan) equipped with a Ti inverted microscope. A Plan Apo VC DIC N2 dry objective lens (Nikon, Tokyo, Japan; numerical aperture, NA = 0.75) was adopted for imaging, with the refractive index of the imaging medium set to 1.00 and a pixel calibration of 0.62 μm per pixel. Images were captured at a resolution of 1024 × 1024 pixels with a bit depth of 12 bits. Galvanometer scanning mode was used, and a DU4 dual-channel synchronous detector was used to collect fluorescence signals simultaneously. Synchronized dual-channel scanning was performed for FITC and mCherry, and both channels were excited by 488.0 nm and 561.0 nm lasers. The pinhole radius was uniformly set to 46.86 μm to ensure consistent optical section thickness for the two channels. For the FITC and mCherry channels, the emission wavelengths were 525.0 nm and 595.0 nm, laser powers were 10.2% and 20.0%, photomultiplier tube gain settings were 75 and 88, and baseline offsets were −13 and −10, respectively. Identical imaging parameters were applied to both normoxic and hypoxic groups, and ImageJ software (version 1.54p) was used to quantify the fluorescence intensity of the captured images.

### 2.5. Transmission Electron Microscopy

To characterize the cellular ultrastructure, cells underwent primary fixation in 2.5% glutaraldehyde within 0.1 M phosphate buffer (pH 7.4) at 4 °C for 2 h, followed by secondary fixation using 1% osmium tetroxide for 1 h. After dehydration in a graded ethanol series, the dehydrated cell pellets were subjected to infiltration and embedded in Epon 812 resin. Ultrathin sections with a thickness of 70 nm were cut using a Leica UC7 ultramicrotome (Leica Microsystems, Wetzlar, Germany). The sections were double-stained with uranyl acetate and lead citrate, fully air-dried, and subsequently imaged under a JEM-1400 transmission electron microscope (JEOL, Akishima, Japan).

### 2.6. RT-qPCR

Total RNA was extracted using Trizol reagent (Invitrogen, Thermo Fisher Scientific, Waltham, MA, USA) and reverse-transcribed using PrimeScript RT Master Mix (Takara, Kusatsu, Shiga, Japan). Quantitative PCR was performed using SYBR Green Master Mix (Takara, Kusatsu, Shiga, Japan). Primer sequences were purchased from Aoke Dingsheng Biotechnology (Beijing, China): *Bnip3* forward 5′-AGGTTTTCCTTCCATCTCTGTTACTG-3′, reverse 5′-TGTGTGAACAGAAGTCAGATCCAAA-3′; *Bnip3l* forward 5′-AGACTGGTCCAGTAGACCCGAA-3′, reverse 5′-CAGCACATGAGAAAGGAAGAGAGA-3′; *Pink1* forward 5′-GTCCTGAAGGGAGCAGACG-3′, reverse 5′-TTAAGATGGCTTCGCTGGAG-3′; *Fundc1* forward 5′-CGAGTATTTGGCCACAGTTCC-3′, reverse 5′-CCACTGTGACTGGCAACCTG-3′; *Atg5* forward 5′-AAGAGGAGCCAGGTGATGATTC-3′, reverse 5′-ACTCAAGGTGGTTCCATCTAGC-3′; *Actb* forward 5′-CCCATCTATGAGGGTTACGC-3′, reverse 5′-TTTAATGTCACGCACGATTTC-3′. Relative expression was calculated using the 2^−ΔΔCt^ method with *Actb* as the internal control. All RT-qPCR data represent three or four independent biological replicates.

### 2.7. Statistical Analysis

Statistical analyses of all experimental data were performed using SPSS 20.0 software. All experiments were carried out with at least three independent biological replicates (*n* = 3 per group). Measurement data are expressed as mean ± standard deviation (mean ± SD). Comparisons between the two groups at multiple time points were conducted via two-way analysis of variance (ANOVA) with Holm–Sidak’s multiple-comparison test. Two-tailed independent sample *t*-tests were also applied for intergroup comparisons of other indicators at the same time point. Differences with *p* < 0.05 were considered statistically significant. GraphPad Prism 8.0 software was used for image visualization and plotting.

## 3. Results

### 3.1. Hypoxia Induces Progressive Mitochondrial Protein Degradation and Structural Damage

**Figure 1:**
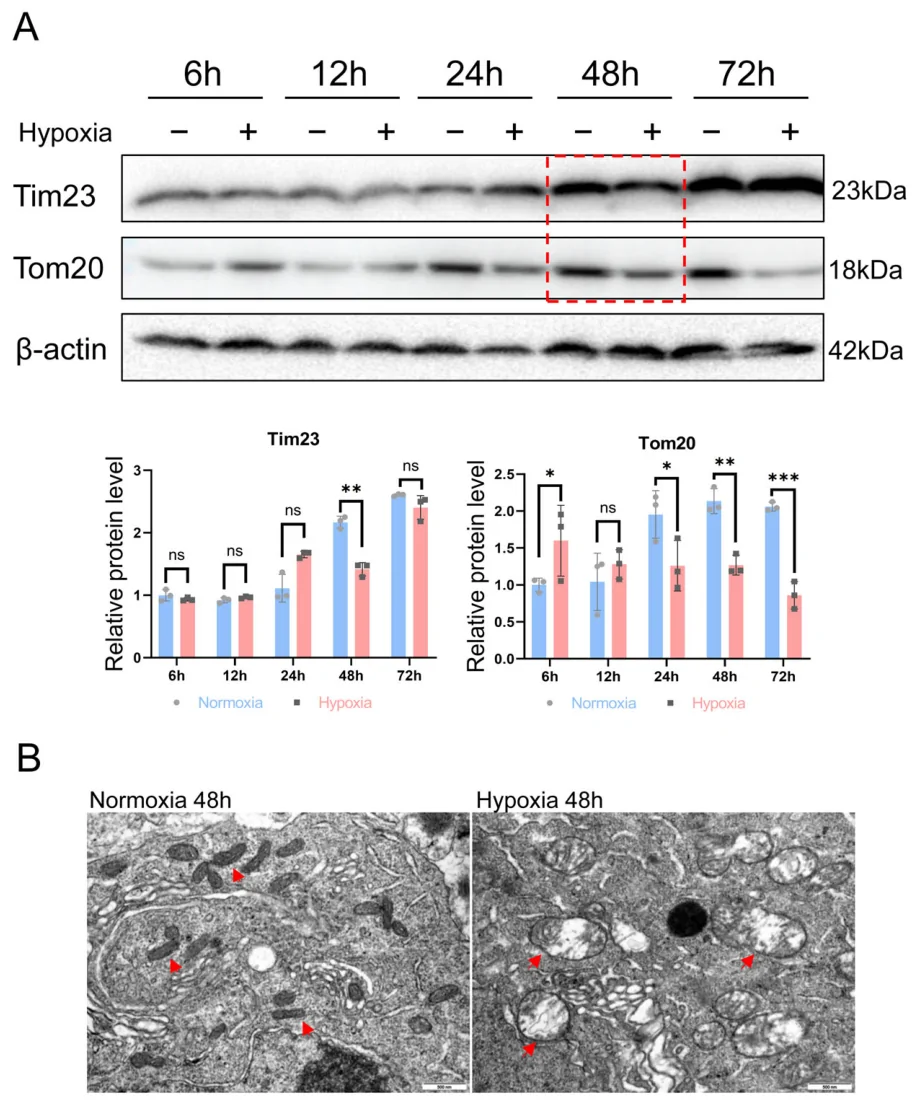
Hypoxia induces mitochondrial protein degradation in HT22 immortalized hippocampal neurons: (**A**) Western blot analysis of mitochondrial resident proteins Tim23 and Tom20 at 6, 12, 24, 48, and 72 h post-hypoxic exposure (*n* = 3). Dashed square indicates mitochondrial proteins with significantly reduced expression after 48 h of hypoxic exposure. (**B**) Transmission electron microscopy of HT22 immortalized hippocampal neurons (*n* = 3). Red arrows indicate mitochondria. Scale bar = 500 nm. Data are presented as mean ± SD. ns, not significant; * *p* ≤ 0.05, ** *p* ≤ 0.01, *** *p* ≤ 0.001. All original uncropped Western blot images are available in Supplementary Materials.

To establish whether hypoxia compromises mitochondrial integrity in hippocampal neurons, we examined the expression of mitochondrial resident proteins Tim23 (inner membrane) and Tom20 (outer membrane) over a 72-h time course. Western blot analysis with biological replicates (*n* = 3) revealed distinct temporal patterns. Tom20 levels exhibited a modest upregulation at 6 h (*p* < 0.05) and progressively decreased at 24 h (*p* < 0.05), 48 h (*p* < 0.01), and 72 h (*p* < 0.001) under hypoxia conditions. Tim23 showed a significant reduction at 48 h (*p* < 0.01), returning to baseline by 72 h (Figure 1A). This time-dependent, protein-specific degradation pattern suggests progressive mitochondrial clearance rather than acute nonspecific damage, with the outer membrane protein Tom20 being more persistently affected. Transmission electron microscopy revealed mitochondrial swelling, cristae disruption and fragmentation, and matrix vacuolization in 48 h hypoxia-exposed neurons compared to the dense cristae and intact membranes observed under normoxic conditions (Figure 1B).

### 3.2. Hypoxia Induces Mitochondrial Fragmentation

**Figure 2:**
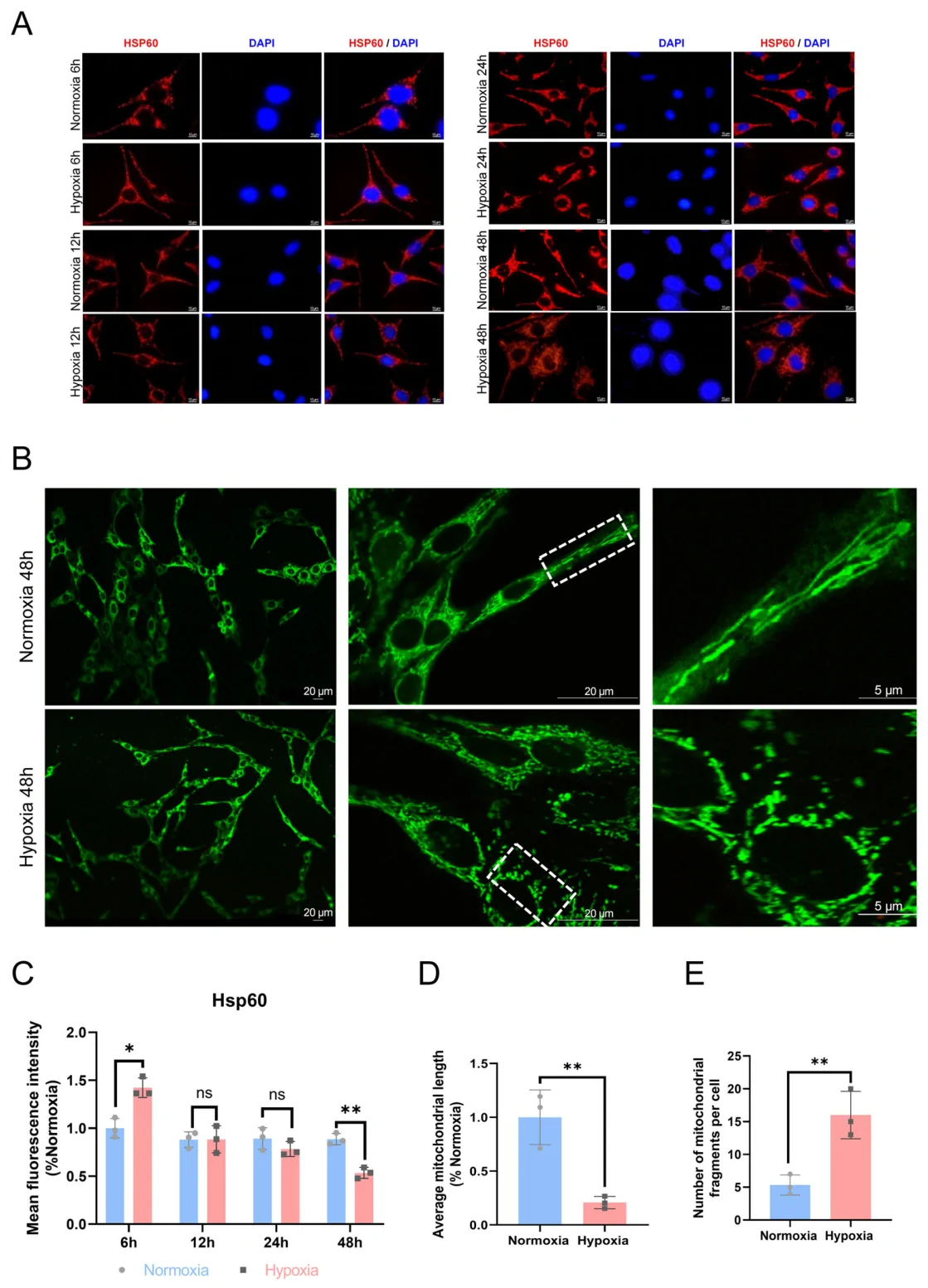
Hypoxia induces mitochondrial fragmentation in HT22 immortalized neurons: (**A**) Immunofluorescence for HSP60 (red) under normoxic and hypoxic conditions for 48 h (*n* = 3). Scale bars: 10 μm. (**B**) MitoTracker (green) labeling under normoxic and hypoxic conditions at 48 h (*n* = 3). Scale bars: left, 20 μm; middle, 20 μm; right, 5 μm. Dashed square indicates magnified cells. (**C**) Quantification of HSP60 fluorescence intensity from (**A**) under normoxic and hypoxic conditions for 48 h (*n* = 3). (**D**) Average mitochondrial length quantified from (**B**) in normoxia and hypoxia groups at 48 h (*n* = 3). (**E**) Number of mitochondrial fragments per cell quantified from (**B**) in normoxia and hypoxia groups at 48 h (*n* = 3). Data are presented as mean ± SD. ns, not significant; * *p* ≤ 0.05, ** *p* ≤ 0.01.

To assess hypoxia-induced mitochondrial alterations, HT22 cells were immunostained with HSP60, a mitochondrial matrix protein, after normoxic or hypoxic (1% O_2_) exposure (Figure 2A). Quantitative analysis revealed that HSP60 fluorescence intensity exhibited a biphasic response—upregulation (*p* < 0.05) at 6 h, recovery to baseline at 12–24 h, and significant depletion (*p* < 0.01) at 48 h (Figure 2C)—further suggesting progressive loss of the mitochondrial mass under sustained hypoxic stress. Live-cell MitoTracker labeling was performed to explore the mitochondrial morphological shift (Figure 2B). After 48 h of hypoxic exposure, morphometric analysis further revealed severe mitochondrial fragmentation, characterized by a significant reduction (*p* < 0.01) in average mitochondrial length (Figure 2D) and a significant increase (*p* < 0.01) in the number of mitochondrial fragments per cell (Figure 2E). These observations suggest that hypoxia disrupts mitochondrial dynamics and promotes mitochondrial fragmentation in HT22 immortalized hippocampal neurons.

### 3.3. Hypoxia Triggers Mitophagy in HT22 Immortalized Neurons

**Figure 3:**
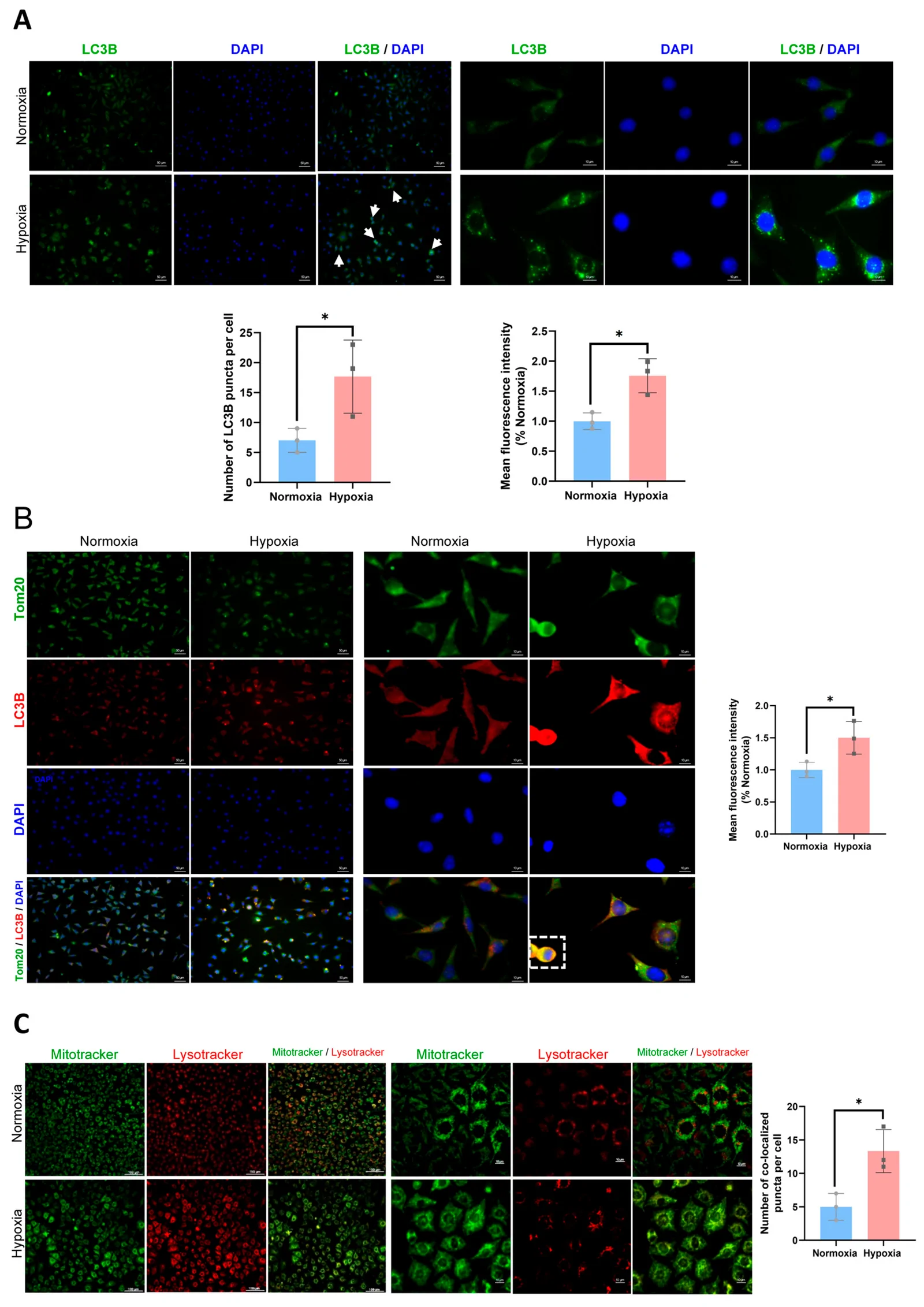
Hypoxia triggers mitophagy in HT22 immortalized neurons: (**A**) Immunofluorescence for LC3B under normoxic and hypoxic (48 h) conditions (*n* = 3). Scale bars: left, 50 μm; right, 10 μm. White arrows indicate LC3B-positive puncta. (**B**) Dual immunofluorescence for LC3B (green) and Tom20 (red) under normoxic and hypoxic conditions. Yellow puncta indicate colocalization (*n* = 3). Scale bars: left, 50 μm; right, 10 μm. Dashed boxes indicate LC3B/Tom20 colocalization regions. (**C**) Dual immunofluorescence for MitoTracker (green) and LysoTracker (red) at 48 h (*n* = 3). Arrowheads indicate mitochondria–lysosome apposition/fusion under hypoxia. Scale bars: left, 100 μm; right, 10 μm. Data are presented as mean ± SD. * *p* ≤ 0.05.

Having observed mitochondrial damage and fragmentation, we further examined HT22 cells after 48 h of hypoxic exposure for changes in autophagy-related markers. Under normoxic conditions, LC3B exhibited diffuse cytoplasmic distribution with sparse punctate structures. By 48 h, hypoxic exposure was associated with a marked increase in LC3B puncta (Figure 3A). LC3B puncta were sparsely distributed under normoxia but densely populated under hypoxia. Quantitative analysis revealed that the number of LC3B puncta per cell (*p* < 0.05) and the mean fluorescence intensity (*p* < 0.05) were significantly higher in the hypoxic group than in the normoxic group. At higher magnification, individual puncta displayed distinct spherical morphology, a feature consistent with autophagosome-like structures.

To examine the spatial relationship between LC3B puncta and mitochondria, we performed dual immunofluorescence for LC3B and Tom20. Under normoxia, LC3B and Tom20 showed largely separate distributions at both magnifications. Following 48 h of hypoxic exposure, increased colocalization between LC3B puncta and Tom20-positive mitochondria was observed (Figure 3B). Quantitative analysis confirmed a significant increase in the fluorescence intensity of colocalized regions (*p* < 0.05), consistent with the presence of LC3B-positive structures in proximity to mitochondria.

To examine whether mitochondria and lysosomes showed altered spatial proximity, we assessed their colocalization at 48 h. Colocalized mitochondria–lysosome structures were observed in both groups. Quantitative analysis showed that the number of colocalized mitochondria–lysosome puncta per cell was significantly higher (*p* < 0.05) in the hypoxic group. As shown in the magnified image, dual labeling revealed increased membrane apposition between mitochondria and lysosomes under hypoxic conditions (Figure 3C), a feature that may precede autophagosome–lysosome fusion but does not by itself demonstrate the fusion event.

Taken together, these findings document morphological changes in HT22 cells following hypoxic exposure, including increased LC3B puncta, enhanced LC3B–mitochondria colocalization, and increased mitochondria–lysosome apposition. While these features are compatible with the engagement of mitophagy-related pathways, we acknowledge that the current data do not establish mitophagic flux. LC3B puncta accumulation may equally reflect impaired autophagic degradation, and two-dimensional colocalization does not demonstrate mitochondrial delivery into the lysosomal lumen or subsequent breakdown. Therefore, our conclusions are limited to morphological evidence supporting further investigation, rather than a definitive demonstration of functional mitophagy.

### 3.4. RT-qPCR Screening Reveals a Multi-Pathway Mitophagic Network Featuring Bnip3/Bnip3l

**Figure 4:**
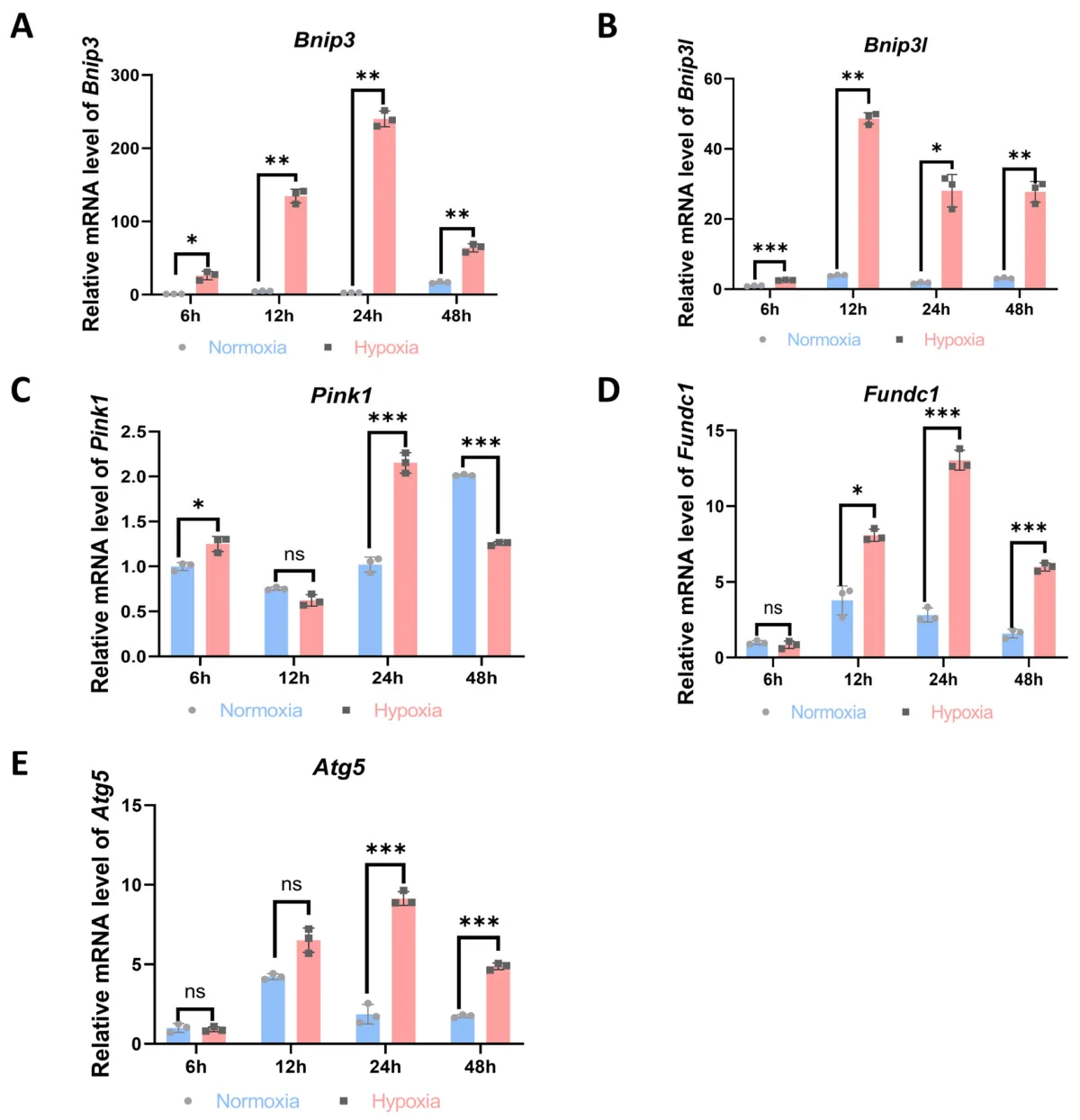
RT-qPCR screening reveals a multi-pathway autophagic network in HT22 immortalized neurons, as shown by analysis of (**A**) *Bnip3*, (**B**) *Bnip3l*, (**C**) *Pink1*, (**D**) *Fundc1*, and (**E**) *Atg5* (*n* = 3–4). Data are presented as mean ± SD. ns, not significant; * *p* ≤ 0.05, ** *p* ≤ 0.01, *** *p* ≤ 0.001.

Having observed morphological evidence consistent with mitophagy, we next screened for transcriptional changes in candidate genes that may contribute to this response. We performed a comprehensive screening of five key autophagy-related genes encompassing hypoxia-specific receptors (*Bnip3*, *Bnip3l*), canonical damage sensors (*Pink1*), alternative hypoxia-responsive receptors (*Fundc1*), and general autophagy machinery (*Atg5*). *Bnip3* demonstrated the most robust transcriptional response, with significant elevation detected as early as 6 h (*p* < 0.05) and peak expression at 24 h (*p* < 0.01), followed by upregulation at 48 h (*p* < 0.01) (Figure 4A). *Bnip3l* showed parallel kinetics with comparable magnitude, with early upregulation at 6 h (*p* < 0.001), peak expression at 12 h (*p* < 0.01), and sustained elevation at 24 h (*p* < 0.05) and 48 h (*p* < 0.01) (Figure 4B). In contrast, *Pink1* mRNA exhibited slight upregulation at 6 h (*p* < 0.05), no significant change at 12 h, and modest upregulation at 24 h (*p* < 0.001), but paradoxical downregulation at 48 h (*p* < 0.001) (Figure 4C), suggesting a transient or secondary transcriptional response under prolonged hypoxic stress. *Fundc1* mRNA expression displayed moderate induction with delayed onset, showing no significant change at 6 h, upregulation at 12 h (*p* < 0.05), and peak elevation at 24 h (*p* < 0.001) (Figure 4D), consistent with its established role as a hypoxia-responsive mitophagy receptor. Notably, the general autophagy machinery gene *Atg5* mRNA expression followed similar delayed kinetics, with no significant change at 6 h and 12 h, peak expression at 24 h (*p* < 0.001), and significant elevation at 48 h (*p* < 0.001) (Figure 4E), indicating that hypoxia activates both selective mitophagy and the broader autophagic apparatus in a temporally coordinated manner. Comparative analysis revealed that *Bnip3* and *Bnip3l* showed the earliest significant induction and the highest fold change, preceding and exceeding *Pink1*, *Fundc1*, and *Atg5* activation at the transcriptional level. This temporal and amplitude hierarchy suggests that *Bnip3*/*Bnip3l* represent the most prominent early-transcriptional responders in this hypoxic neuronal model, while acknowledging that mRNA abundance does not necessarily reflect functional protein activity or pathway dominance. Importantly, these transcriptional changes establish an association with hypoxia rather than a causal role in mediating mitophagy, and future loss-of-function studies are required to determine whether BNIP3 or BNIP3L functionally contributes to the observed phenotypes.

### 3.5. BNIP3 and BNIP3L Protein Upregulation and Location

**Figure 5:**
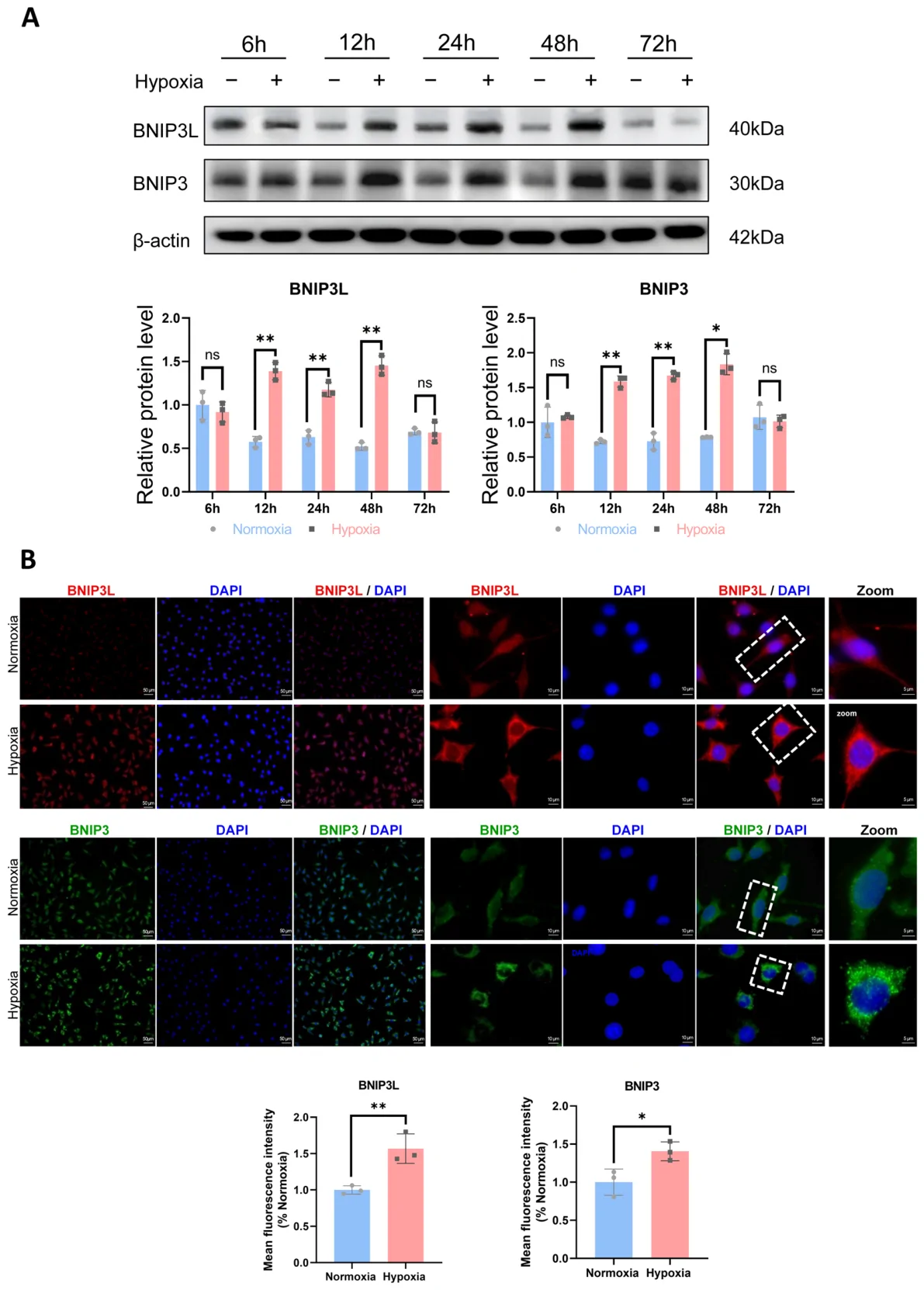
BNIP3 and BNIP3L protein upregulation and preferential accumulation in neuronal processes: (**A**) Western blot of BNIP3 and BNIP3L protein at 6–72 h post-hypoxia (*n* = 3). (**B**) Immunofluorescence for BNIP3 (green) and BNIP3L (red) under normoxic and hypoxic (48 h) conditions (*n* = 3). Scale bars: left, 50 μm; middle, 10 μm; right, 5 μm. Dashed square indicates cells in the zoomed-in region. Data are presented as mean ± SD. ns, not significant; * *p* ≤ 0.05; ** *p* ≤ 0.01.

To validate the RT-qPCR screening findings, we examined BNIP3L protein expression using Western blot across three independent biological replicates. As shown in Figure 5A, hypoxia induced a significant, time-dependent upregulation of BNIP3 and BNIP3L in HT22 immortalized hippocampal neurons. BNIP3L protein levels were markedly elevated relative to normoxic controls at 12 h (*p* < 0.01) and 24 h (*p* < 0.01), with a modest yet significant increase that persisted at 48 h (*p* < 0.01), whereas BNIP3 protein levels were also markedly elevated relative to normoxic controls at 12 h (*p* < 0.01) and 24 h (*p* < 0.01), with a modest but significant increase sustained at 48 h (*p* < 0.05). Notably, this upregulation was transient: BNIP3 and BNIP3L expression returned to near-baseline levels by 72 h (*p* > 0.05), suggesting a time-restricted activation of the BNIP3/BNIP3L-associated mitophagy pathway during hypoxic adaptation.

Immunofluorescence staining further revealed that BNIP3 and BNIP3L were localized in the nucleus and cytoplasm, with expression also observed along neuronal processes in HT22 cells under normoxic and hypoxic conditions (Figure 5B). Notably, zoomed-in views (5 μm scale) revealed that hypoxia-induced BNIP3 adopted a granular morphology, whereas BNIP3L appeared more filamentous and widely dispersed throughout the cytoplasmic space. Quantitative immunofluorescence analysis revealed that the mean fluorescence intensity of both BNIP3L (*p* < 0.01) and BNIP3 (*p* < 0.05) was significantly increased in the hypoxic group compared with the normoxic group. Collectively, these findings demonstrate that hypoxia upregulates BNIP3 and BNIP3L expression, with their distinct morphological configurations suggesting potentially non-redundant roles in the hypoxic autophagic response.

## 4. Discussion

While hypoxia is a well-established trigger for mitophagy across various cell types, the specific pathways involved and their relative contributions are highly context-dependent [21]. This study provides an initial characterization of the molecular events associated with hypoxia-induced mitophagy in HT22 immortalized hippocampal neurons, revealing a temporally coordinated program characterized by the upregulation of BNIP3 and BNIP3L.

### 4.1. Temporal Progression of Mitochondrial Damage

Our findings demonstrate that in hippocampal neurons, chronic hypoxia (1% O_2_) initiates a progressive degradation of mitochondrial inner and outer membrane proteins, Tim23 and Tom20, which is consistent with the concept that mitochondrial integrity is compromised as a prerequisite for autophagic recognition [22]. From a broader perspective, the degradation of outer mitochondrial membrane proteins, including Tom20, is mediated by Parkin via the ubiquitin–proteasome system, independent of the autophagic machinery [23]. Additionally, prolonged hypoxia inhibits mitochondrial biogenesis and diminishes mitochondrial mass, leading to progressively decreased expression of both Tim23 and Tom20 relative to normoxic levels, with each protein displaying differential temporal dynamics [24]. This degradation was accompanied by ultrastructural damage, including mitochondrial swelling, cristae disruption, and matrix vacuolization, aligning with reports that hypoxic stress induces profound mitochondrial morphological alterations in other systems, such as cardiomyocytes and pulmonary arterial cells [25,26]. The observed mitochondrial fragmentation evidenced by HSP60 immunostaining and MitoTracker labeling is a critical step that likely facilitates the engulfment of damaged organelles by autophagosomes. Indeed, hypoxia-induced mitochondrial fission has been linked to Drp1 activation through pathways involving HIF-1α and ERK/PKA signaling in primary hippocampal neurons [27], and our observations of fragmented mitochondria in HT22 cells are in agreement with these mechanistic studies.

### 4.2. Mitophagy-Associated Phenotypes: Evidence and Limitations

Mitophagy serves as a key quality control mechanism for eliminating damaged mitochondria and has been reported to be activated under chronic hypoxic conditions in various cell types [25,28]. In HT22 cells, hypoxic exposure was associated with morphological changes consistent with engagement of the mitophagy pathway, including increased LC3B puncta, LC3B–Tom20 colocalization, and mitochondria–lysosome apposition. However, these fluorescence-based colocalization data do not distinguish between enhanced mitophagic flux and impaired autophagosome/lysosome turnover, nor do they demonstrate mitochondrial delivery into lysosomes or subsequent degradation. Thus, while our findings are compatible with the hypothesis that hypoxia recruits mitophagy-related machinery, definitive demonstration of functional mitophagy—for instance, via lysosomal inhibitor-based LC3-II/I and p62 turnover assays—will require future studies [29].

### 4.3. A Multi-Pathway Autophagic Network in the Cellular Response to Hypoxia

Our RT-qPCR screening identified a multi-pathway autophagic network with transcriptional changes associated with hypoxia, with *Bnip3* and *Bnip3l* representing the most prominent early-transcriptional responders, exhibiting the earliest and most robust transcriptional upregulation. While *Pink1*, *Fundc1*, and *Atg5* were also significantly induced at the transcriptional level, their lower amplitude and delayed kinetics suggest that they may function as auxiliary or parallel components. This hierarchy is particularly noteworthy given the established role of the PINK1/Parkin pathway in mitophagy. In many cell types, such as cardiomyocytes subjected to hypoxia/reoxygenation, PINK1/Parkin signaling is a dominant mechanism for mitochondrial clearance [30,31]. However, in our neuronal model, the PINK1/Parkin pathway showed a relatively delayed and lower-amplitude transcriptional response, suggesting a potentially secondary role. This divergence is consistent with studies demonstrating that in response to hypoxia or hypoxia-mimetic agents, damaged mitochondria can be removed via a Parkin-independent mechanism that relies on BNIP3 and NIX (BNIP3L) [32]. Furthermore, research on hypoxic–ischemic brain injury in neonatal rats has shown that both PINK1/Parkin-dependent and -independent pathways (including NIX and FUNDC1) are upregulated, with the temporal dynamics suggesting a shift in pathway dominance [33]. Our data, showing a delayed and lower-amplitude induction of *Pink1*, align with the concept that in hippocampal neurons under chronic, moderate hypoxia, *Bnip3*/*Bnip3l* may represent the predominant receptor candidates transcriptionally, whereas the PINK1/Parkin pathway may be more critical under delayed stress conditions or may function as a compensatory mechanism at the transcriptional level.

The transcriptional upregulation of *Bnip3* and *Bnip3l* was paralleled by changes at the protein level, with BNIP3 and BNIP3L protein expression showing a progressive increase from 12 to 48 h of hypoxia. Quantitative analysis of mean immunofluorescence intensity revealed upregulation of BNIP3L and BNIP3 proteins in the hypoxic group at 48 h. This protein upregulation is critical, as BNIP3 and BNIP3L are mitophagy receptors that directly recruit LC3 to the mitochondrial surface, thereby initiating autophagosome formation [34,35]. The HIF-1α/BNIP3 axis is a well-characterized pathway in hypoxic cells, and our findings reinforce its importance in the nervous system [36,37]. A notable observation from the immunofluorescence analysis was the divergent subcellular patterns of BNIP3 and BNIP3L following 48 h of hypoxic exposure. Under hypoxic conditions, the granular appearance of BNIP3 puncta contrasted markedly with the filamentous morphology of BNIP3L, suggesting that these two structurally homologous mitophagy receptors may adopt distinct spatial configurations within overlapping cytoplasmic compartments during the hypoxic autophagic response.

The role of FUNDC1, another hypoxia-responsive mitophagy receptor, was also examined. While *Fundc1* mRNA expression was significantly upregulated, its induction was not as pronounced as that of *Bnip3*/*Bnip3l*. This is consistent with studies showing that FUNDC1-mediated mitophagy can be regulated by other factors, such as MARCH5, which ubiquitinates and degrades FUNDC1 to fine-tune the mitophagic response under hypoxia [38]. In our model, the moderate transcriptional upregulation of *Fundc1* suggests it may play a supporting role, potentially acting in concert with BNIP3/BNIP3L, although functional cooperation remains to be tested. The concurrent transcriptional activation of *Atg5* indicates that the general autophagy machinery is also primed, which is necessary for the formation of autophagosomes that will engulf the mitochondria targeted by the receptors [39].

The significance of our findings lies in the initial characterization of a *Bnip3*/*Bnip3l*-associated transcriptional program in hippocampal neurons, which contrasts with the PINK1-dominant paradigm observed in some other neural and non-neural systems. For instance, in dopaminergic neurons, CK2 signaling has been shown to positively induce PINK1/Parkin-dependent mitophagy in response to rotenone [40]. Our study highlights the cell-type and stress-specific nature of mitophagy pathways. Furthermore, our use of a chronic, 1% O_2_ hypoxia model over 48 h is more representative of clinical conditions such as chronic cerebral ischemia or chronic high-altitude hypoxia exposure, as opposed to acute anoxia or chemical hypoxia [33,41]. Hypoxia may trigger adaptive responses, including metabolic reprogramming and potentially enhanced mitophagy, to promote cell survival [42]. The temporal profile of *Bnip3*/*Bnip3l* transcriptional upregulation, with an early onset at 6 h, positions them as “early-responder” genes that initiate a sustained response. This is in line with the role of BNIP3 in mediating cellular adaptation to prolonged hypoxic stress, such as in follicular development, where HIF-1α/BNIP3 signaling protects granulosa cells from apoptosis [36]. A recent study demonstrated that in Tibetan hypoxic adaptation, the combination of EPAS1 downregulation and the M9a haplogroup activates HIF-1α/BNIP3/NIX-mediated mitophagy via ROS, thereby alleviating hypoxia-induced inflammation and apoptosis and enhancing cellular hypoxia tolerance [43]. This observation suggests that HIF-1α may function upstream of BNIP3L/BNIP3 in the cellular response to prolonged hypoxia, potentially as part of a protective mechanism. Concurrently, the inhibition of oxidative phosphorylation (OXPHOS) was associated with the activation of the mitophagy pathway under chronic hypoxia [44]. However, our current data do not distinguish whether the observed mitophagy is initiated by OXPHOS failure or through HIF-dependent activation; this represents a key question for future mechanistic studies.

### 4.4. Functional Context from the Existing Literature

Our previous laboratory findings have demonstrated that hypoxic exposure induces enhanced programmed cell death, impaired mitochondrial respiratory chain, reduced mitochondrial ATP production, and increased ROS levels in hippocampal tissue and neurons, concomitant with decreased spine density [3,5,6]. Increased neuronal apoptosis in the hippocampus has also been observed in other studies [41]. Exposure to hypoxia in primary rat hippocampal neurons resulted in increased ROS generation, along with suppressed ATP production, collapsed MMP, and decreased neuronal survival [27]. Importantly, these studies provide functional corroboration that extends the conclusions of our study. Nevertheless, the involvement of mitophagy in hypoxic hippocampal neuronal injury should not be simplistically interpreted as excessive mitophagy-driven pathological damage. On the one hand, our study lacks knockout and overexpression experiments to definitively address this issue; on the other hand, protective roles of mitophagy have been documented in other models of hypoxia [32].

### 4.5. Study Limitations and Perspectives

Despite the novel insights provided by this study, several limitations must be acknowledged. First, all experiments were conducted using the HT22 immortalized murine hippocampal neuronal cell line. While this model is valuable for mechanistic studies, it lacks the complex cellular microenvironment of the intact brain, including interactions with glial cells, endothelial cells, and the influence of dynamic blood flow. The physiological role of BNIP3/BNIP3L in vivo may be modulated by these factors. For example, astrocytes, which are critical for neuronal support, also express BNIP3 under hypoxia, and their response could indirectly affect neuronal survival [45]. Second, this study is descriptive and correlative; we have not performed functional gain- or loss-of-function experiments to directly establish a causal role for BNIP3/BNIP3L in the observed mitophagy and its impact on neuronal viability. Future studies employing siRNA-mediated knockdown or overexpression of these genes are essential to confirm their necessity and sufficiency. Additionally, we acknowledge that the lack of direct hypoxic marker validation, systematic cell viability characterization, and membrane potential measurement limits the interpretative scope of the present findings, and these aspects warrant further investigation. Third, we did not perform a quantitative assessment of autophagic flux, for example, by monitoring LC3B-II/I conversion and p62 turnover in the presence of lysosomal inhibitors such as chloroquine. Such experiments would provide a more quantitative assessment of autophagic flux and thus represent a key direction for future research to complement the descriptive nature of the present study [29]. Additionally, the precise structural domains mediating the interaction between BNIP3/BNIP3L and LC3B were not investigated. Understanding these molecular details could provide targets for therapeutic intervention.

In summary, this study establishes a foundational understanding of the molecular choreography of hypoxia-induced mitophagy in hippocampal neurons, highlighting its association with BNIP3/BNIP3L and providing a framework for future translational studies aimed at preserving neuronal function under hypoxic stress.

## 5. Conclusions

In conclusion, this descriptive study provides a temporal framework for hypoxia-induced mitophagy in HT22 immortalized hippocampal neuronal cells. *Bnip3* and *Bnip3l* exhibit the earliest and most pronounced transcriptional upregulation among five screened autophagy-related genes under prolonged hypoxia (1% O_2_), paralleled by BNIP3 and BNIP3L protein elevation and associated with mitochondrial fragmentation, increased LC3B puncta, and increased mitochondria–lysosome proximity. The preferential accumulation of BNIP3 and BNIP3L with distinct granular and filamentous morphologies suggests spatially patterned quality control responses. These findings generate the hypothesis that BNIP3/BNIP3L-associated pathways may contribute to hypoxic mitochondrial clearance in neurons, which requires causal validation through gain- and loss-of-function experiments and functional assessment of cell viability, bioenergetics, and oxidative stress.

## Data Availability

The original contributions presented in this study are included in the article/Supplementary Materials. Further inquiries can be directed to the corresponding authors.

## Supplementary Materials

The following supporting information can be downloaded at https://www.mdpi.com/article/10.3390/biology15181659/s1, Supplementary Figures S1–S7: Original uncropped Western blot images for Figure 1 and Figure 5.
