# Supplementary material for: Hypoxia Triggers BNIP3/BNIP3L-Associated Mitophagy in HT22 Immortalized Hippocampal Neurons: A Temporal Descriptive Study
Source: Biology (Basel). 2026 Sep 19;15(18):1659. doi: 10.3390/biology15181659 (PMC13604653; doi:10.3390/biology15181659)
Supplement: Supplementary file 1 [file biology-15-01659-s001.zip › biology-4469404-supplementary.pdf]

Supplementary Materials

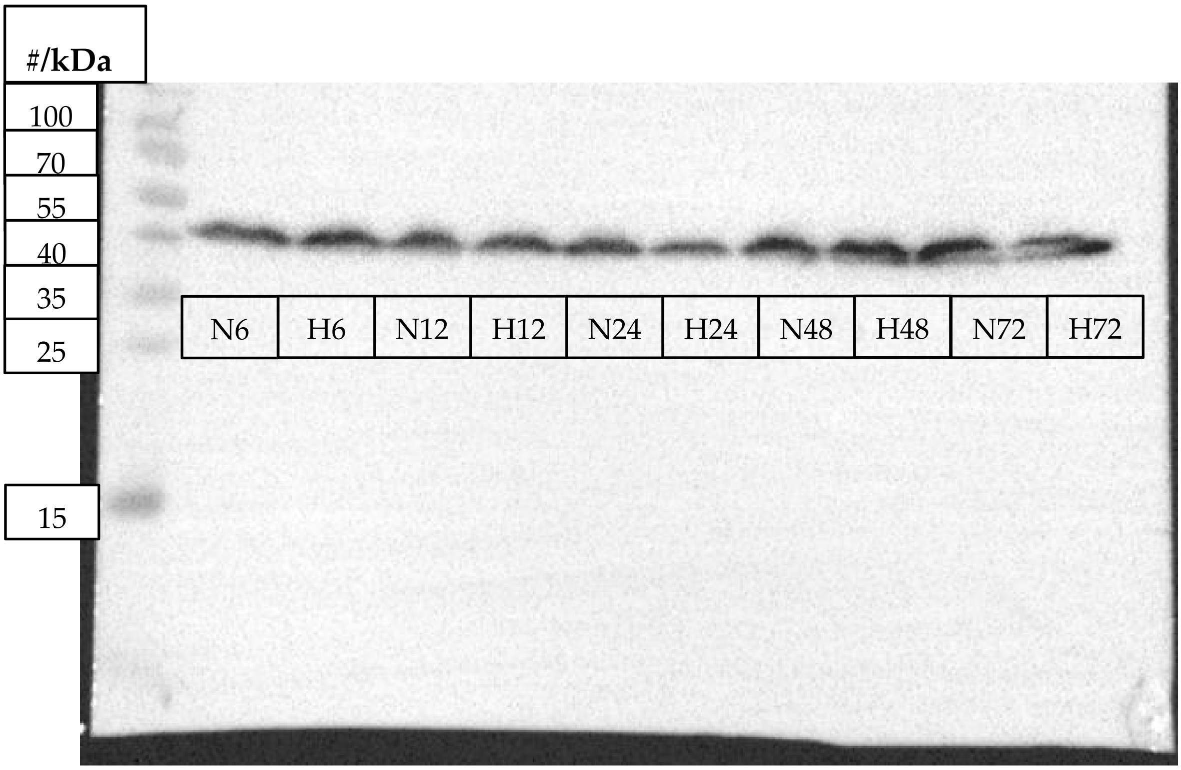

Figure S1. Western blot membrane of β-actin(~42 kDa) protein detected with anti-β -actin (ab8226 ; 1:2000; Abcam, Cambridge, UK) antibody. Gel-separated proteins were transferred to PVDF membranes (0.22 µm pore size; Bio-Rad, Hercules, USA) by semi-dry electroblotting (1.5 mA per cm^2, 50 min). Membranes, incubated with a horseradish peroxidase-conjugated secondary antibody (7076S ; 1:2000; Cell Signaling Technology, Danvers, MA, USA), were developed with ECL Substrate (Mishu Biotech, Xi'an, Shaanxi, China). #Weight marker (molecular weight in kDa): Thermo Scientific™/PageRuler™ Prestained Protein Ladder, 10 to 180 kDa; catalogue number: 26616. Blot images, prior to the densitometry readings, were converted to grayscale with ImageJ (ImageJ v.1.53, National Institutes of Health, Maryland, USA) as follows: Image -> Type –> 8 bit, next: Image -> Adjust–> Brightness/Contrast –> Auto. N, normoxia; H, hypoxia, at 6, 12, 24, 48, and 72 h of exposure.

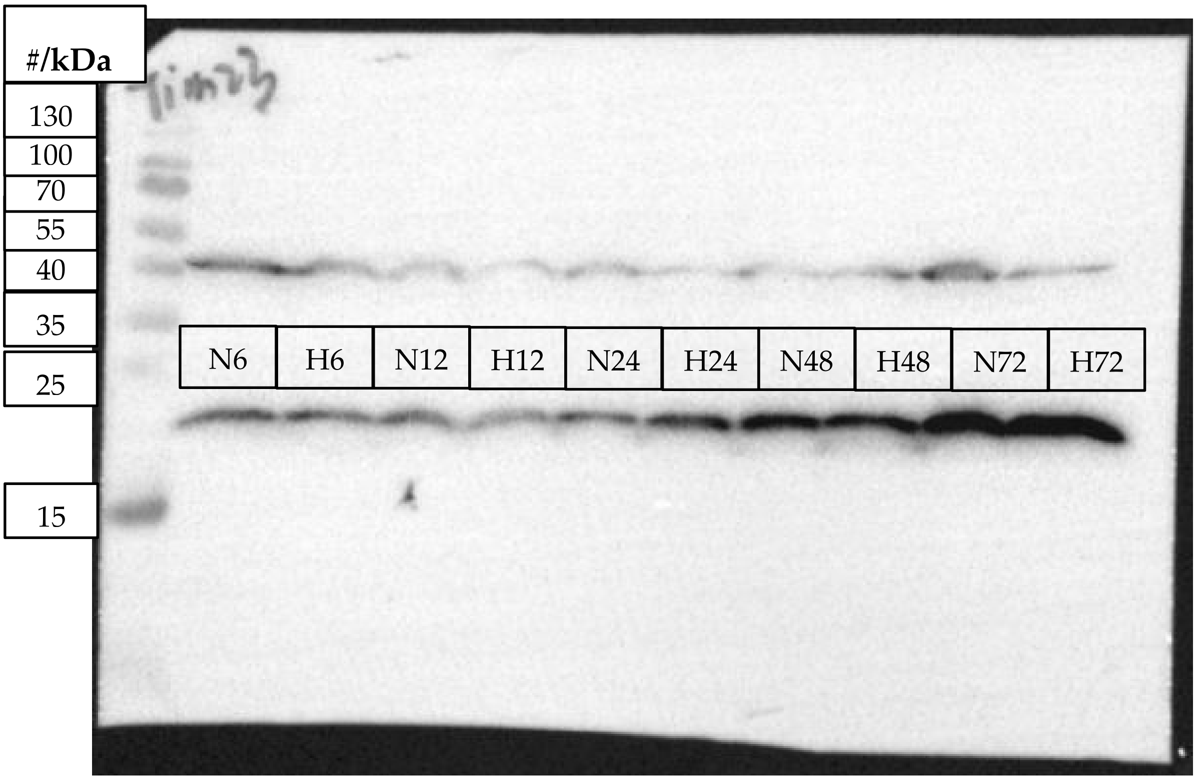

Figure S2. Western blot membrane of Tim23(~23 kDa) protein detected with anti-Tim23 (BD611222; 1:1000; BD Biosciences, San Jose, CA, USA) antibody. Gel-separated proteins were transferred to PVDF membranes (0.22 μm pore size; Millipore, Billerica, MA, USA) by semi-dry electroblotting (1.5 mA per cm^2, 50 min). Membranes, incubated with a horseradish peroxidase-conjugated secondary antibody (7076S ; 1:2000; Cell Signaling Technology, Danvers, MA, USA), were developed with ECL Substrate (Mishu Biotech, Xi'an, Shaanxi, China). #Weight marker (molecular weight in kDa): Thermo Scientific™/PageRuler™ Prestained Protein Ladder, 10 to 180 kDa; catalogue number: 26616. Blot images, prior to the densitometry readings, were converted to grayscale with ImageJ (ImageJ v.1.53, National Institutes of Health, Maryland, USA) as follows: Image -> Type –> 8 bit, next: Image -> Adjust–> Brightness/Contrast –> Auto. N, normoxia; H, hypoxia, at 6, 12, 24, 48, and 72 h of exposure.

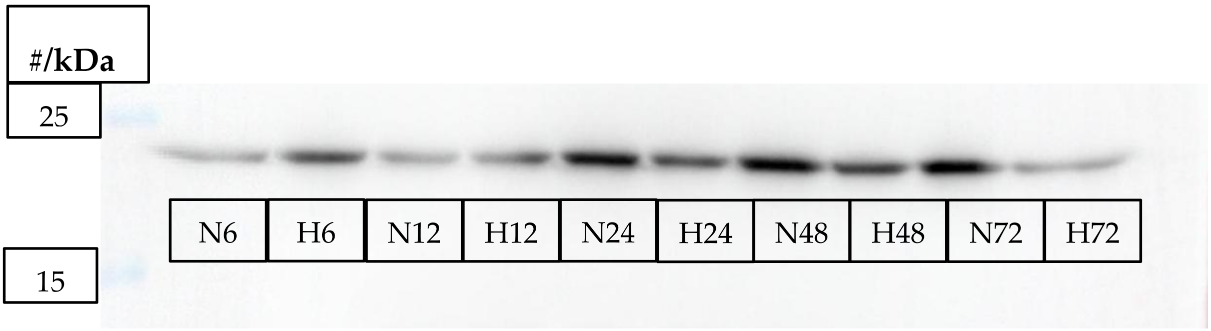

Figure S3. Western blot membrane of Tom20(~18 kDa) protein detected with anti-Tom20 (BD612278 ; 1:2000; BD Biosciences, San Jose, CA, USA) antibody. Gel-separated proteins were transferred to PVDF membranes (0.22 μm pore size; Millipore, Billerica, MA, USA) by semi-dry electroblotting (1.5 mA per cm^2, 50 min). Membranes, incubated with a horseradish peroxidase-conjugated secondary antibody (7076S;1:2000; Cell Signaling Technology, Danvers, MA, USA), were developed with ECL Substrate (Mishu Biotech, Xi'an, Shaanxi, China). #Weight marker (molecular weight in kDa): Thermo Scientific™/PageRuler™ Prestained Protein Ladder, 10 to 180 kDa; catalogue number: 26616. Blot images, prior to the densitometry readings, were converted to grayscale with ImageJ (ImageJ v.1.53, National Institutes of Health, Maryland, USA) as follows: Image -> Type –> 8 bit, next: Image -> Adjust–> Brightness/Contrast –> Auto. N, normoxia; H, hypoxia, at 6, 12, 24, 48, and 72 h of exposure.

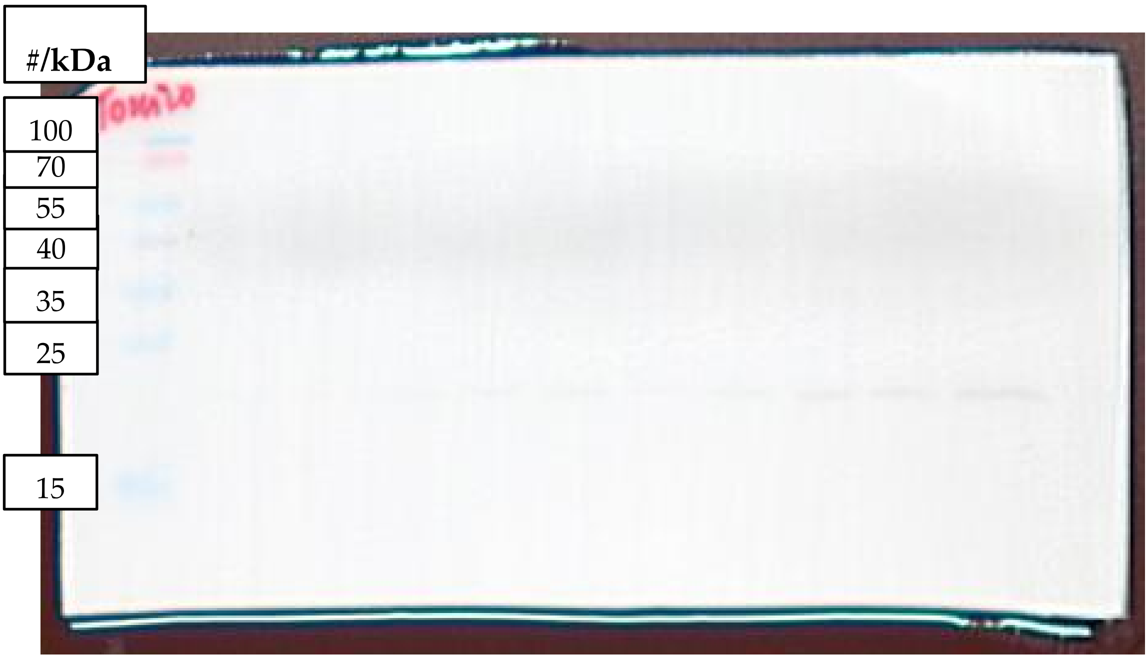

Figure S4. Western blot membrane of Tom20(~18 kDa) protein detected with anti-Tom20 (BD612278 ; 1:2000; BD Biosciences, San Jose, CA, USA) antibody. (Display location only). Gel-separated proteins were transferred to PVDF membranes (0.22 μm pore size; Millipore, Billerica, MA, USA) by semi-dry electroblotting (1.5 mA per cm^2, 50 min). Membranes, incubated with a horseradish peroxidase-conjugated secondary antibody (7076S; 1:2000; Cell Signaling Technology, Danvers, MA, USA), were developed with ECL Substrate (Mishu Biotech, Xi'an, Shaanxi, China). #Weight marker (molecular weight in kDa): Thermo Scientific™/PageRuler™ Prestained Protein Ladder, 10 to 180 kDa; catalogue number: 26616.

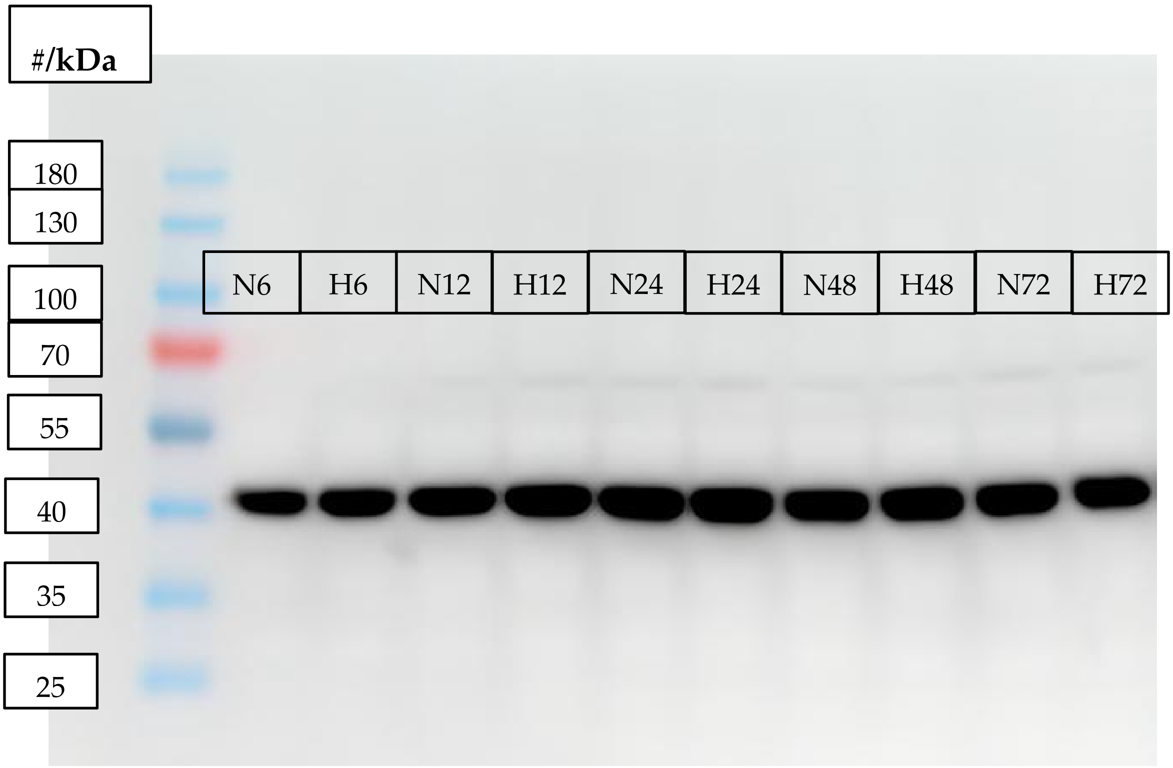

Figure S5. Western blot membrane of β-actin(~42 kDa) protein detected with anti-β -actin (ab8226 ; 1:2000; Abcam, Cambridge, UK) antibody. Gel-separated proteins were transferred to PVDF membranes (0.22 μm pore size; Millipore, Billerica, MA, USA) by semi-dry electroblotting (1.5 mA per cm^2, 50 min). Membranes, incubated with a horseradish peroxidase-conjugated secondary antibody (7076S ; 1:2000; Cell Signaling Technology, Danvers, MA, USA), were developed with ECL Substrate (Mishu Biotech, Xi'an, Shaanxi, China). #Weight marker (molecular weight in kDa): Thermo Scientific™/PageRuler™ Prestained Protein Ladder, 10 to 180 kDa; catalogue number: 26616. Blot images, prior to the densitometry readings, were converted to grayscale with ImageJ (ImageJ v.1.53, National Institutes of Health, Maryland, USA) as follows: Image -> Type –> 8 bit, next: Image -> Adjust–> Brightness/Contrast –> Auto. N, normoxia; H, hypoxia, at 6, 12, 24, 48, and 72 h of exposure.

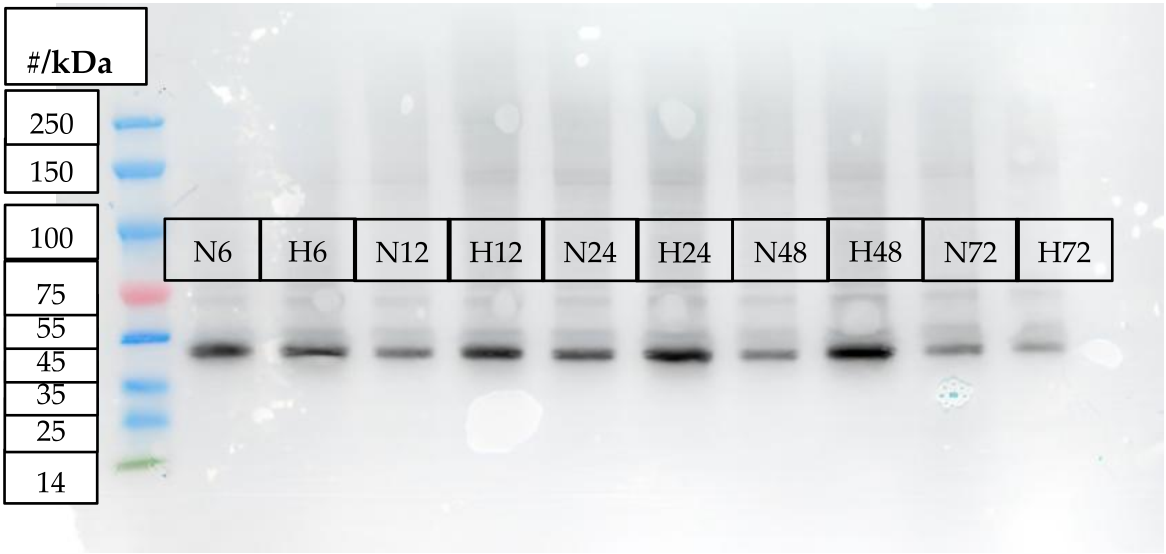

Figure S6. Western blot membrane of BNIP3L(~40 kDa) protein detected with anti-BNIP3L (ab8399 ; 1:500; Abcam, Cambridge, UK) antibody. Gel-separated proteins were transferred to PVDF membranes (0.22 μm pore size; Millipore, Billerica, MA, USA) by semi-dry electroblotting (1.5 mA per cm^2, 50 min). Membranes, incubated with a horseradish peroxidase-conjugated secondary antibody (7074S ; 1:2000; Cell Signaling Technology, Danvers, MA, USA), were developed with ECL Substrate (Mishu Biotech, Xi'an, Shaanxi, China). #Weight marker (molecular weight in kDa): NcmColor™ Color Prestained Protein Ladder, 14 to 250 kDa; catalogue number: P9006. Blot images, prior to the densitometry readings, were converted to grayscale with ImageJ (ImageJ v.1.53, National Institutes of Health, Maryland, USA) as follows: Image -> Type –> 8 bit, next: Image -> Adjust–> Brightness/Contrast –> Auto. N, normoxia; H, hypoxia, at 6, 12, 24, 48, and 72 h of exposure.

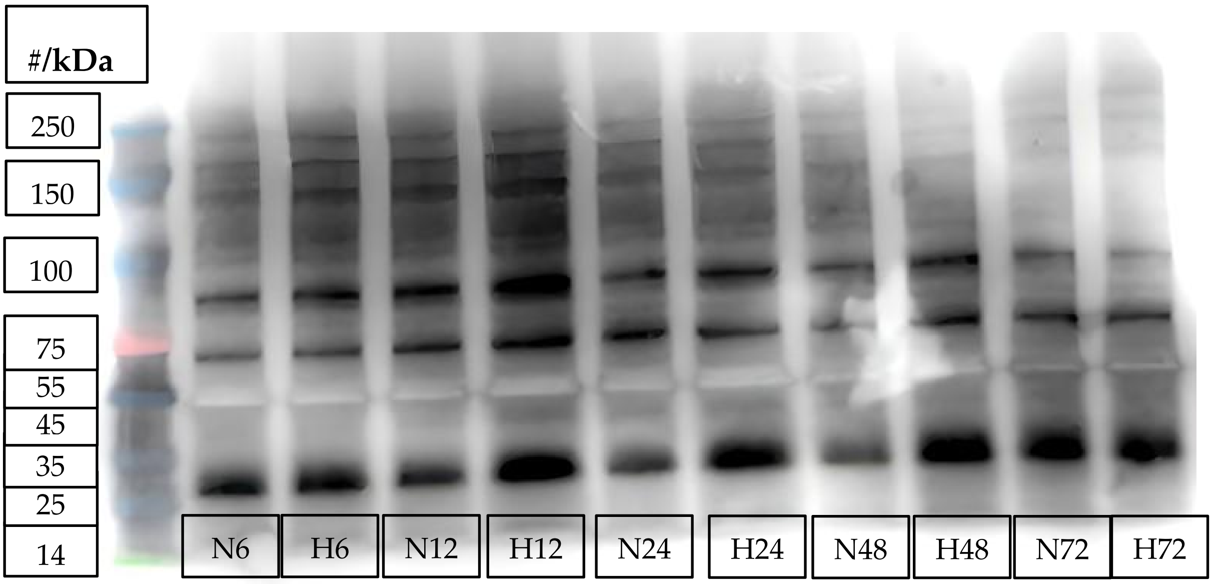

Figure S7. Western blot membrane of BNIP3(~30kDa) protein detected with anti-BNIP3 (ab10433 ; 1:1000; Abcam, Cambridge, UK) antibody. Gel-separated proteins were transferred to PVDF membranes (0.22 μm pore size; Millipore, Billerica, MA, USA) by semi-dry electroblotting (1.5 mA per cm^2, 50 min). Membranes, incubated with a horseradish peroxidase-conjugated secondary antibody (7076S ; 1:2000; Cell Signaling Technology, Danvers, MA, USA), were developed with ECL Substrate (Mishu Biotech, Xi'an, Shaanxi, China). #Weight marker (molecular weight in kDa): NcmColor™ Color Prestained Protein Ladder, 14 to 250 kDa; catalogue number: P9006. Blot images, prior to the densitometry readings, were converted to grayscale with ImageJ (ImageJ v.1.53, National Institutes of Health, Maryland, USA) as follows: Image -> Type –> 8 bit, next: Image -> Adjust–> Brightness/Contrast –> Auto. N, normoxia; H, hypoxia, at 6, 12, 24, 48, and 72 h of exposure.
